# Antimicrobial pharmacodynamics of vancomycin and disulfiram (Antabuse®) in *Staphylococcus aureus*

**DOI:** 10.3389/fmicb.2022.1092257

**Published:** 2023-01-06

**Authors:** Hasitha Chavva, Yogesh Meka, Timothy E. Long

**Affiliations:** ^1^Department of Pharmaceutical Science and Research, School of Pharmacy, Marshall University, Huntington, WV, United States; ^2^Department of Biomedical Sciences, Joan C. Edwards School of Medicine, Marshall University, Huntington, WV, United States

**Keywords:** vancomycin, disulfiram (Antabuse®), pharmacodynamics, synergy, methicillin-resistant *Staphylococcus aureus*, VISA

## Abstract

**Introduction:**

Intravenous vancomycin (VAN) is the primary treatment for systemic infections due to methicillin-resistant *Staphylococcus aureus* (MRSA). Pharmacokinetic/pharmacodynamic target (PK/PD) indices for VAN therapies are more difficult to achieve for MRSA isolates with a minimum inhibitory concentration (MIC) greater than 1 µg mL^-1^. This research investigated the *in vitro* antimicrobial PD interaction of disulfiram (DSF) with VAN as a potential adjuvant therapy for infections due to these bacteria.

**Methods:**

The antimicrobial interaction was assessed by differential analysis using checkerboard titration testing, time-kill studies, flow cytometry, and the post-antibiotic effect (PAE) experiment. Ten MRSA strains with MICs ranging from 1 to >256 µg mL^-1^ for VAN were evaluated. A comprehensive PD assessment of the VAN/DSF interaction was performed using the VAN-intermediate (VISA) strain Mu50 (MIC 8 µg mL^-1^).

**Results:**

The addition of DSF lowered the MIC and minimum bactericidal concentration (MBC) of VAN in either a synergistic or additive manner for the MRSA panel. Optimal bactericidal effects and suppression of VISA Mu50 growth were observed with a 4/8 µg mL^-1^ combination of VAN/DSF, but not the individual drugs. Flow cytometry further confirmed the enhanced killing action on a cellular level; however, the addition of DSF had an overall antagonistic effect on the PAEs for VAN.

**Discussion:**

This research established that DSF exhibits additive to synergistic killing action with VAN for MRSA. Conversely, antagonism was observed on the PAE of VAN with DSF addition for the Mu50 strain. Flow cytometry further confirmed the enhanced bactericidal effect on a cellular level while revealing that DSF may counteract the muropeptide fortification mechanism against VAN in VISA.

## 1. Introduction

Antibiotic resistance is a global public health concern in human and veterinary medicine (Watkins and Bonomo, 2020). Breakpoints based on either minimum inhibitory concentration (MIC) or zone of inhibition values are used in both resistance surveillance and pharmacotherapy to establish whether a bacterial isolate is susceptible, moderately susceptible (i.e., intermediate), or resistant to an antibiotic (Turnidge and Paterson, 2007). In pharmacotherapy, clinicians further rely on pharmacokinetic (PK) and pharmacodynamic (PD) data to guide the administration of intravenous antibiotics for deep-seated infections. Such infections may require treatment with two antibiotics capable of attaining PK/PD target indices (e.g., AUC/MIC) recommended by clinical practice guidelines when combined (Levison and Levison, 2009).

Combination therapies have been sought as a means to treat systemic infections by methicillin-resistant *Staphylococcus aureus* (MRSA) isolates that exhibit an intermediate level of vancomycin (VAN) resistance (VISA: MIC 4–8 μg mL^−1^) (Gomes et al., 2015). For infections involving VAN-susceptible *S. aureus* (VSSA: MIC ≤2 μg mL^−1^), clinical guidelines recommend an AUC/MIC ≥400 to avoid resistance development and ensure adequate tissue penetration (Rybak et al., 2020). While achievable for many MRSA infections, PK/PD target indices are more difficult to attain for *S. aureus* isolates with a VAN MIC >1 μg mL^−1^ that may necessitate the use of an alternative treatment (Lodise et al., 2008; Jeffres, 2017; Rybak et al., 2020). Moreover, such infections are at higher risk for VAN therapy failure particularly in regions where the MIC “creep” is thought to exist (Steinkraus et al., 2007). Reasons for this gradual rise in MICs may include altered crosslinked peptidoglycan levels and increases in muropeptides binding targets to consume VAN, as detected in VISA isolates (Hiramatsu et al., 2014).

In an effort to discover agents that can lower the MIC of VAN in MRSA, a recent drug library screening identified the alcohol sobriety aid disulfiram (Antabuse®) as a potential antibiotic adjuvant (Moore et al., 2020). Strain-dependent, summative fractional inhibitory concentrations (ΣFICs) values of ≤0.5 to 1 for VAN/DSF co-treatments were further reported in studies conducting preliminary isobologram analyses, indicating synergistic to additive interactions (Long, 2017; Frazier et al., 2019; Moore et al., 2020). Other groups have also recently found that the initial metabolite of DSF, diethyldithiocarbamate, has synergistic potential with VAN in the presence of Cu^2+^ against MRSA biofilms (Kaul et al., 2022) and other infectious agents (Kaul et al., 2021). With these findings, a more detailed assessment of the antimicrobial PD interactions of VAN/DSF in *S. aureus* was merited. The following research describes the effect of VAN/DSF on MRSA growth and survival over time with a focus on strains having intermediate levels of VAN resistance.

## 2. Materials and methods

### 2.1. Synergism studies

Isobologram analysis of DSF and VAN synergism was performed with 10 MRSA strains (i.e., two VSSA, one hVISA, six VISA, and one VRSA) using the microdilution checkerboard assay in a 6 × 10 matrix format (Moody, 1992; Supplementary Table S1). Early log-phase inoculums were prepared by combining 1 mL of overnight broth cultures with 9 mL of fresh broth and grown to an OD_600_ of 0.6–0.8. The cultures were standardized to a 0.5 McFarland saline suspension and diluted to 10^5^ cfu mL^−1^ in cation-adjusted Mueller-Hinton broth (CAMHB). Flat bottom microtiter plates containing two-fold serial dilutions of VAN (range 0.125–32 μg mL^−1^) and DSF (range 1–16 μg mL^−1^) were then inoculated and incubated in a water-jacketed incubator at 37°C. Sustained MICs were recorded for individual and combined treatments that conferred visual growth inhibition after 48 h. The minimum bactericidal concentrations (MBCs) were obtained by inoculating 4 μL samples from each well on Mueller-Hinton agar (MHA) to identify treatments that yielded no growth following 48 h incubation. The fractional inhibitory and bactericidal concentrations (FIC/FBC) were calculated by dividing the MIC or MBC of the VAN/DSF treatment by the MIC or MBC of either DSF or VAN alone. The lowest summative calculated from the FIC/FBC values was interpreted according to indices: synergism ≤0.5 (++); additive 0.5 < to 1 (+); indifferent 1 < to 4 (±); antagonism 4 < (−) (Doern, 2014).

### 2.2. Growth studies

Growth studies by colony forming unit (cfu) analysis were performed in duplicate using six MRSA strains comprised of hVISA (Mu3), VISA (Mu50, ADR-217, ADR-219, ADR-220), and VRSA (VRSA-MI) isolates (Aeschlimann et al., 1999). Early log-phase cultures with a target inocula of 10^5^ cfu mL^−1^ obtained from 0.5 McFarland saline suspensions were treated with antibiotic(s) ranging from 1 to 16 μg mL^−1^ or equal volume of vehicle (DMSO). Cultures of 10^6^ and 10^7^ cfu mL^−1^ were similarly tested to compare the effect of inoculum size in standard CAMHB test media. Moreover, CAMHB containing 5% fetal-bovine serum (FBS) was used to determine the influence of serum proteins and Hank’s balanced salt solution (HBSS) supplemented with 1% glucose was used to establish whether non-dividing bacteria were susceptible to the treatments.

The cultures were incubated with shaking (150 rpm) at 37°C following treatment. At each time point, 10-fold serial dilutions of bacteria in saline were spread on Mueller-Hinton agar (MHA) plates. The plates were incubated at 37°C for 24 h and the number of cfu mL^−1^ were recorded as the mean of two replicates. Treatments were characterized as bactericidal if there was ≥3 log_10_ cfu mL^−1^ reduction (i.e., ≥99.9%) and bacteriostatic if the decrease was 0 to <3 log_10_ cfu mL^−1^ compared to the initial inoculum (Lee and Burgess, 2013). Synergism was further defined as a decrease of ≥2 log_10_ cfu mL^−1^ for VAN/DSF when compared to most active agent alone (Lee and Burgess, 2013). Additive and indifferent effects were similarly defined as reductions of 1 to 2 log_10_ cfu mL^−1^ and 0 to <1 log_10_ cfu mL^−1^, respectively.

To measure the effect on VISA Mu50 growth over time by turbidity measurements, 100 μL of vehicle- (DMSO) or drug-treated cultures with an inoculum size of 5.5 × 10^5^ cfu mL^−1^ were dispensed in a flat bottom 96-well plate and incubated at 37°C for 48 h. Optical density (OD) readings were recorded on a Molecular Devices SpectraMax® 384 plate reader at 600 nm following 5 s agitation. The same cultures were used to monitor growth by cfu mL^−1^ counts at time points 0, 2, 4, 8, 24, and 48 h. Growth curves were plotted against time from the mean OD_600_ and cfu values using Prism 9.0.2 software (GraphPad Software, Inc.).

To measure the effect of Mu50 viability over time, the Promega BacTiter-Glo™ Microbial Cell Viability Assay was used to measure intracellular ATP. A 10^5^ cfu mL^−1^ of inoculum of early-log phase VISA Mu50 in CAMHB was incubated (37°C, 150 rpm) with DSF, VAN, VAN/DSF, or equal volume of vehicle (DMSO). At different time points, 1 mL of the treated cultures was chilled on ice for 1 min, combined with Lysing Matrix B 0.1 mm spherical silica beads (MP Biomedicals), and homogenized using a BeadBug™ 6 (Benchmark Scientific) for 5 × 0.5 min at 4350 rpm. Samples of 40 μL were combined with 60 μL of assay reagent in a black 96-well plate and mixed for 1 min in the dark prior to measuring relative luminescence units (RLU) using a Molecular Devices FilterMax™ F3 Plate Reader. Bacterial samples were standardized based on ratio of RLU and protein obtained by combining 200 μL Pierce™ BCA Protein Assay reagent with 100 μL of lysate. Viability curves were plotted against time from the mean data of three replicates.

### 2.3. Flow cytometry studies

VISA Mu50 inoculums of 5.5 × 10^5^ cfu mL^−1^ in 1 mL of CAMHB were treated with either DSF, VAN, VAN+DSF, or DMSO (null). Following incubation (37°C, 22 h, 150 rpm), samples of 100 μL were combined with 100 μL PBS and stained with 1 μL of 1.5 mM propidium iodide (PI, Thermo Fisher Scientific™) and 0.5 mM SYTOX Green (Invitrogen™). For positive controls of >99.9% permeated cells, 5 μL of 10 mM cetyltrimethylammonium bromide (CTAB) was added to a Mu50 null sample. The bacteria were incubated in the dark for 15 min and analyzed on an ACEA Novocyte 2000R flow cytometer equipped with a 488 nm excitation laser and 530/30 (green) and 675/30 (red) emission filters for detection of SYTOX Green (SYTOX) and PI, respectively. Forward scatter (FSC) and side scatter (SSC) plot comparison of unstained cells was used to calibrate the acquisition gate. For each sample, *ca.* 20,000 events were collected and plotted using NovoExpress 1.3.0 software (Agilent Technologies, Inc.). The same method was applied for muropeptide binding assessment using 0.1 μg mL^−1^ Vancomycin, BODIPY™ FL Conjugate (Invitrogen™) in place of SYTOX. At 22 and 44 h time points, *ca.* 50,000 events were collected and plotted using NovoExpress 1.3.0 software. Statistical analysis was conducted by two-way analysis of variance (ANOVA) followed by a Tukey’s multiple-comparison test using Prism 9.0.2 software.

### 2.4. Post-antibiotic effect studies

Durations for the post-antibiotic effect (PAE) was determined using 10^6^ cfu mL^−1^ of early log-phase *S. aureus* inoculums that were incubated with shaking (150 rpm) at 37°C in 5 mL CAMHB containing the antibiotic(s) or vehicle (Pankuch and Appelbaum, 2006). After 2 h, the cultures were diluted 1:1,000 and incubated at 37°C. Bacterial viability was then assessed over time from the initial time point (T_0_) using the drop plate method on MHA (Herigstad et al., 2001). Following 24 h incubation, the time for cfu counts to increase by 1 log_10_ was determined. The PAE was calculated as *T* – *C* where *T* is the difference in time for a 1 log_10_ increase in cfu from T_0_ and *C* is the corresponding time for the untreated control.

## 3. Results

### 3.1. Synergism studies

Table 1 provides the MIC and MBC values for optimal VAN/DSF combinations based on the lowest ΣFIC and ΣFBC calculations derived from isobologram analyses (Moody, 1992). With VSSA strains JE2 and COL, DSF imparted a 50% reduction of VAN MICs in an additive manner (ΣFIC of 0.53–0.75). Similar results were observed for six VISA strains (ΣFIC of 0.5–0.75) exhibiting a VAN of 8 μg mL^−1^. It was noteworthy that the addition of 2–4 μg mL^−1^ DSF lowered the MIC of VAN to the VSSA breakpoint in 50% of the VISA strains including Mu50 whose attenuated susceptibility is attributed to a thicken cell wall and muropeptide retention (Sieradzki and Tomasz, 1997; Hanaki et al., 1998; Cui et al., 2000). Although borderline synergy was observed for the VISA variants, the interaction between VAN and DSF was most evident in VAN-resistant *S. aureus*. Multiple combinations with 4 μg mL^−1^ of DSF gave ΣFICs under 0.3 for the *vanA* VRSA-MI isolate with a VAN MIC of 1,024 μg mL^−1^ (Finks et al., 2009). As with VISA Mu50, the MIC of VAN in VRSA-MI was lowered to the VSSA breakpoint value when combined with 4 μg mL^−1^ of DSF.

**Table 1:** Comparison of MIC and MBC values for VAN and/or DSF treatments.

MRSA strain	MIC (μg mL^−1^)			MBC (μg mL^−1^)		
	VAN	DSF	VAN/DSF^a,b^	VAN	DSF	VAN/DSF^a,b^
VSSA JE2	1	16	0.5/4 (+)	1	>16	0.5/4 (+)
VSSA COL	2	16	1/0.5 (+)	2	16	1/0.5 (+)
hVISA Mu3	2	16	1/0.5 (+)	2	16	1/0.5 (+)
VISA Mu50	8	8	2/4 (+)	8	>16	8/8 (±)
VISA AR-216	8	8	2/4 (+)	8	16	4/1 (+)
VISA AR-217	8	8	4/2 (++)	16	16	4/2 (++)
VISA AR-218	8	8	2/2 (++)	8	16	0.5/4 (+)
VISA AR-219	8	8	4/2 (+)	8	>16	2/8 (++)
VISA AR-220	8	8	4/2 (++)	16	>16	4/2 (++)
VRSA-MI	>32	16	2/4 (++)	>32	>16	1/16 (++)

^a^Lowest concentrations in combination.

^b^Synergism (++), additivity (+), indifference (±) and antagonism (−) were determined by summative fractional inhibitory or fractional bactericidal concentration indices (Moody, 1992).

Table 1 further gives optimal MBCs for the 10 member MRSA panel. Reference isolates JE2 (CA-MRSA), COL (HA-MRSA), and the heterogeneous VISA Mu3 (hVISA) exhibited lower VAN MBCs with DSF, indicating that DSF did not antagonize the bactericidal effects of VAN. The addition of DSF also lowered the VAN MBC for five of six VISA strains and VRSA-MI.

### 3.2. Growth studies

Table 2 reports the effects of VAN/DSF treatments on cfu counts after 24 h using an MRSA panel that includes four VISA strains with a VAN MIC of 8 μg mL^−1^. The VISA reference strain Mu50 was used for the initial comprehensive assessment to compare different treatments, inoculum sizes, and culture media. In CAMHB, bactericidal effects (i.e., 99.9% kill) and synergy were observed for VAN/DSF concentrations of 4/8 and 2/16 μg mL^−1^. Co-treatments with 4/4 (99.1%) and 2/8 (89.7%) μg mL^−1^ also decreased Mu50 cfu counts but in a bacteriostatic, additive manner. Increasing the inocula size from 10^5^ to 10^7^ cfu mL^−1^ likewise conferred bacteriostatic effects with the 4/8 μg mL^−1^ combination (59.1%). In HBSS buffer medium, non-dividing Mu50 showed a similar response to the 4/8 μg mL^−1^ performance in CAMHB (99.8%), while 5% fetal bovine serum (FBS) supplement had no antagonistic effects. Additional MRSA strains also exhibited higher susceptibility to the combination (Table 2). Treatment with VAN/DSF 4/8 μg mL^−1^ impaired the growth of VRSA-MI and three other VISA strains to different degrees of effectiveness in mostly a bacteriostatic manner. In hVISA Mu3, the 1/8 μg mL^−1^ mixture conferred bacteriostatic inhibition.

**Table 2:** Effects of VAN, DSF, and combined treatments on cfu mL^−1^ counts after 24 h.

MRSA strain	Medium	Inoculum^a^	μg mL^−1^		cfu mL^−1^ (24 h)			Interaction^a^
			VAN	DSF	VAN	DSF	VAN/DSF	
VISA Mu50	CAMHB	2.5 ± 2.1 × 10^5^	1	8	>10^9^	>10^9^	1.5 ± 0.7 × 10^6^	None
	CAMHB	4.6 ± 1.9 × 10^5^	2	8	>10^9^	>10^9^	4.7 ± 6 × 10^4^	Bacteriostatic (+)
	CAMHB	4.5 ± 0.4 × 10^5^	2	16	>10^9^	2 × 10^5^	3.0 × 10^2^	Bactericidal (++)
	CAMHB	3.0 ± 1.4 × 10^5^	4	4	2.0 × 10^5^	>10^9^	2.5 × 10^3^	Bacteriostatic (++)
	CAMHB	5.5 ± 2.1 × 10^5^	4	8	2.5 ± 2.1 × 10^8^	>10^9^	6.5 ± 0.7 × 10^2^	Bactericidal (++)
	CAMHB	1.5 ± 0.7 × 10^6^	4	8	>10^9^	>10^9^	6.5 ± 4.9 × 10^5^	Bacteriostatic (±)
	CAMHB	1.1 ± 0.21 × 10^7^	4	8	>10^9^	>10^9^	4.5 ± 3.5 × 10^6^	Bacteriostatic (±)
	CAMHB+FBS	5.5 ± 6.3 × 10^5^	4	8	>10^9^	>10^9^	3.0 ± 1.4 × 10^2^	Bactericidal (++)
	HBSS	2.1 ± 1.2 × 10^5^	4	8	2.0 ± 1.4 × 10^4^	1.5 ± 0.7 × 10^3^	4.0 × 10^2^	Bactericidal (++)
VISA ADR-217	CAMHB	2.5 ± 0.7 × 10^5^	4	8	>10^9^	>10^9^	6.5 ± 2.1 × 10^5^	None
VISA ADR-219	CAMHB	2.0 ± 1.3 × 10^5^	4	8	1.3 ± 0.9 × 10^8^	6.0 ± 4.2 × 10^7^	2.0 × 10^4^	Bacteriostatic (+)
VISA ADR-220	CAMHB	3.0 ± 1.4 × 10^5^	4	8	9.0 ± 5.6 × 10^7^	2.5 ± 0.7 × 10^6^	1.0 × 10^3^	Bacteriostatic (+)
VRSA-MI	CAMHB	1.8 ± 2.1 × 10^5^	2	8	>10^9^	>10^9^	3.5 ± 0.7 × 10^5^	None
	CAMHB	1.8 ± 2.1 × 10^5^	4	4	>10^9^	>10^9^	>10^9^	None
	CAMHB	4.5 ± 0.7 × 10^5^	4	8	>10^9^	>10^9^	1.5 ± 2 × 10^5^	Bacteriostatic (±)
hVISA Mu3	CAMHB	2.5 ± 0.7 × 10^5^	1	8	>10^9^	>10^9^	2.5 ± 2.1 × 10^4^	Bacteriostatic (+)

^a^Synergism (++), additivity (+), and indifference (±) (Moody, 1992).

**Figure 1:**
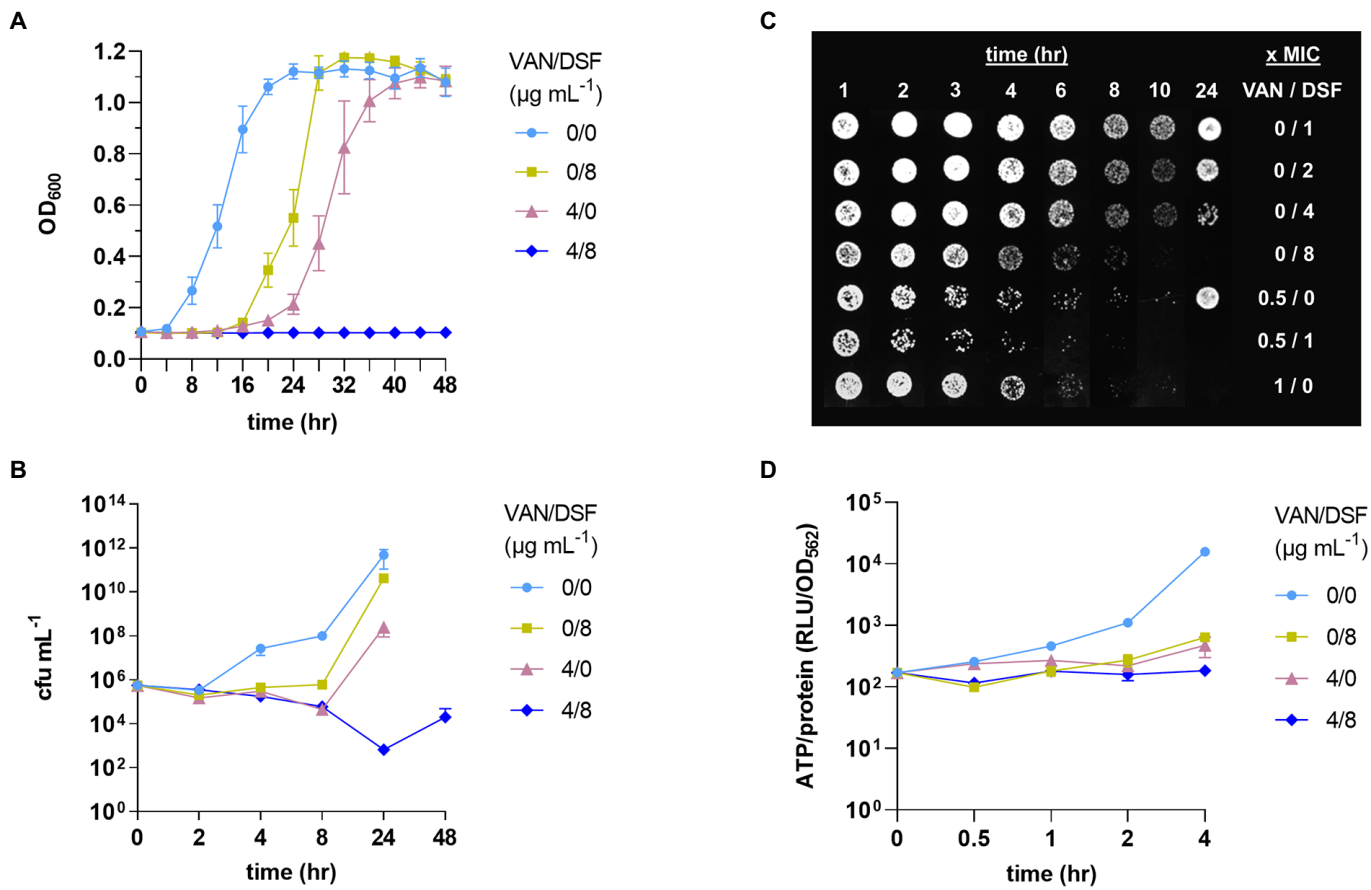
Pharmacodynamic effects of disulfiram (DSF) on VISA Mu50 treated with and without vancomycin (VAN). Treatments were compared by optical growth curves **(A)**, time-kill experiments **(B)**, cell growth on agar after 24 h **(C)**, and measurement of intracellular ATP levels **(D)**.

Kinetics studies were conducted to assess VISA Mu50 response to VAN/DSF over time (Figure 1). Figure 1A reaffirms that DSF lowers the MIC of VAN and continues to suppress optical growth for up to 48 h. In Figure 1B, the time-kill curve reveals that VAN/DSF begins to exhibit bactericidal effects within 4 h and survival continues to decrease over 24 h. Figure 1C confirmed the interaction by showing 1 x MIC DSF and 0.5 x MIC VAN accelerated the reduction of Mu50 growth on agar after an exposure time of 3 to 4 h. Moreover, the results of Figure 1C indicate that DSF has concentration-dependent killing action in Mu50. In a final PD study comparing intracellular ATP levels as a viability measure, VAN/DSF was the lone treatment to suppress energy production over the initial 4 h period (Figure 1D).

### 3.3. Flow cytometry studies

**Figure 2:**
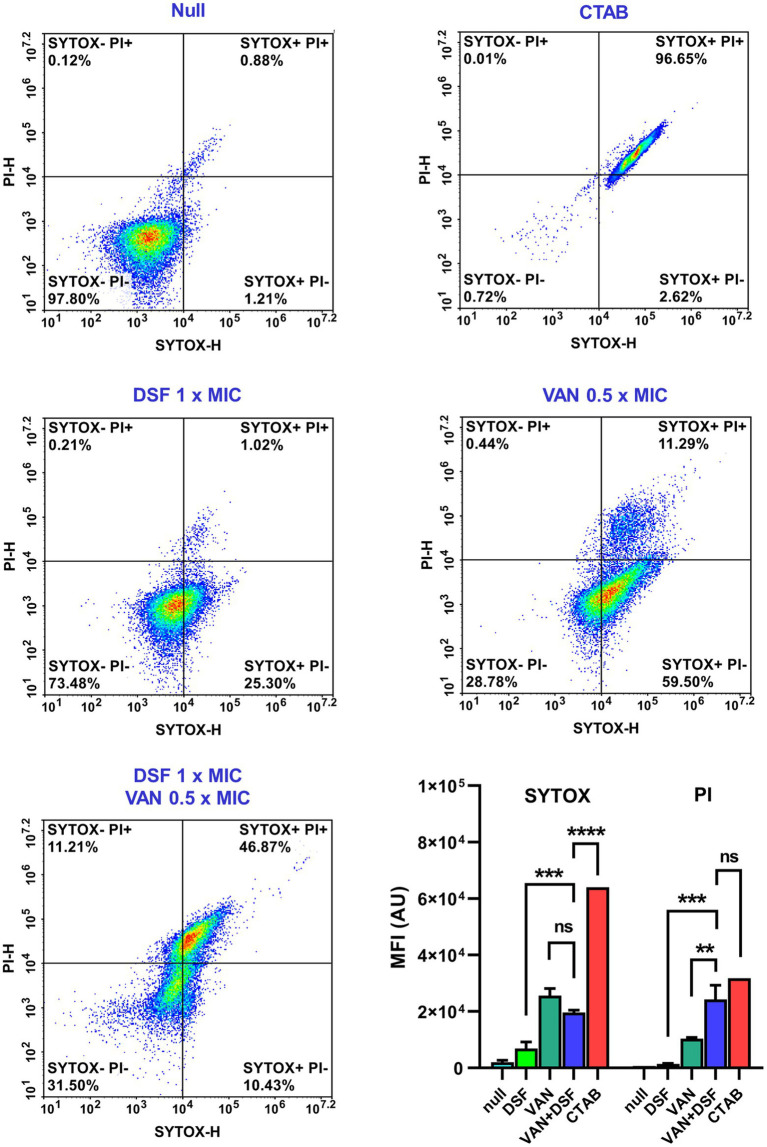
Flow cytometry viability analysis of Mu50 cells stained with SYTOX Green and propidium iodide (PI). Reference plots of bacteria treated with vehicle (null) and detergent (CTAB) were used to distinguish populations of viable (top left) and nonviable (top right) cells. Bar graphs depict the mean fluorescence intensity (MFI) and statistical significance depicted with asterisks (ns = *p* > 0.05; **p ≤ 0.01; ***p ≤ 0.001; ****p ≤ 0.0001).

The antimicrobial PDs of VAN and DSF treatments on VISA Mu50 were further assessed at a cellular level by flow cytometry. A double stain technique with SYTOX (green) and PI (red) was first used to populate nonviable bacteria based on the degree that the two nucleic acid dyes permeate the cell membrane (Roth et al., 1997). The populations were distinguished by reference plots of viable untreated bacteria (null) and nonviable SYTOX^+^/PI^+^ bacteria whose cell membranes were permeated by the detergent CTAB (Figure 2, top). Figure 2 shows a moderate increase in SYTOX-labeled cells with treatments of either DSF or VAN. By comparison, treatment with VAN and DSF gave a denser population of SYTOX^+^/PI^+^ cells, reaffirming that the bactericidal capacity is greater with the combination.

**Figure 3:**
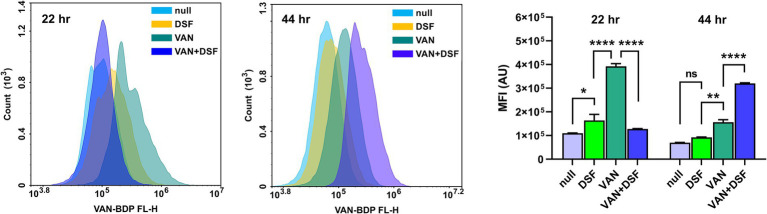
Comparison of flow cytometry histograms and mean fluorescence of Mu50 labeled with VAN-BDP FL following 22 and 44 h treatment with 1 × MIC DSF and/or 0.5 × MIC VAN. Data represents the mean fluorescence intensity (MFI) and statistical significance depicted with asterisks (ns = *p* > 0.05; *p ≤ 0.05; **p ≤ 0.01; ****p ≤ 0.0001).

Flow cytometry further enabled differential analysis of VAN binding to cell wall muropeptides using a BODIPY conjugate of VAN (VAN-BDP FL). In the experiment, Mu50 cultures incubated for 22 and 44 h with either vehicle (DMSO), 1 × MIC DSF and/or 0.5 × MIC VAN were stained and measured for fluorescence *via* the FITC channel. Figure 3 reveals that untreated (null) and DSF-treated cells had similar levels of VAN-BDP FL mean fluorescence intensities (MFIs) after 22 and 44 h. By comparison, cultures receiving VAN alone showed significantly higher VAN-BDP FL binding at both time points. Surprisingly, the 24 h VAN/DSF samples gave comparable MFIs to DSF, possibly revealing that DSF may counteract the fortification mechanism of “false” muropeptide binding targets for VAN in Mu50 (Cui et al., 2000). At the later time point, the VAN/DSF cultures displayed a MFI similar to the 22 h VAN samples, indicating increased muropeptide capturing of VAN-BDP FL (Sieradzki and Tomasz, 1997).

### 3.4. Post-antibiotic effect studies

Clinical practice guidelines for systemic MRSA infections recommend intermittent over continuous IV infusion of VAN in patients with normal renal function (Rybak et al., 2020). The post-antibiotic effect (PAE) of VAN together with its 6 to 12 h half-life (Rybak, 2006) and other factors enable dosing intervals every 8 to 12 h in adult patients. The influence of DSF on the PAE of VAN was the final PD parameter assessed in this study. Table 3 reveals that both VAN and DSF exhibited strain-, concentration-, and time-dependent PAEs. Consistent with prior reports, treatment with 1 x MIC VAN for 1 h showed a PAE of 1.25 h and greater durations after 2 h (Aeschlimann et al., 1999; Suller and Lloyd, 2002). Comparably shorter PAEs were observed for DSF after 1 and 2 h, which was not unexpected due its extensive metabolism as a thiol-reactive drug (Custodio et al., 2022). Likewise, the VAN/DSF gave shorter PAEs than VAN alone in Mu50 and JE2, but not COL, for all combinations at the 1 and 2 h durations.

**Table 3:** Comparison of PAEs following VAN and/or DSF treatments for 1 and 2 h.

MRSA strain	μg mL^−1^		time	PAE (hr)		
	VAN	DSF	(hrs)	VAN	DSF	VAN/DSF
VISA Mu50	4	8	1	0.75	0.25	0.50
	8	8	1	1.25	0.50	0.75
	8	8	2	3.25	0.75	1.25
VSSA COL	2	16	1	0.50	0.50	1.50
	2	16	2	1.25	1.50	1.50
VSSA JE2	1	16	1	1.25	0.75	1.00
	1	16	2	2.25	1.25	1.25

## 4. Discussion

Systemic infections due to MRSA requires immediate intervention to minimize the onset of complications and halt disease progression. Factors affecting clinical responses to anti-MRSA antibiotics have been defined for VAN and include PD elements (Kollef, 2007). Clinical success rates were markedly lower for MRSA bacteremia isolates with VAN MICs ≥1 μg mL^−1^ and reduced rates of VAN clearance (Sakoulas et al., 2004). Compared to other antistaphylococcal agents (e.g., nafcillin), VAN exhibits slower killing action as a time-dependent, concentration-independent antibiotic. The research here revealed that the addition of DSF can accelerate VAN killing of *S. aureus* and lower the MIC/MBC values. As a standalone antimicrobial, DSF displayed concentration-dependent eradication of Mu50 (Figure 1). In clinical practice, a concentration-dependent and a time-dependent killing antibiotic will sometimes be combined due to synergistic killing in tandem. A respective example is the dual use of an aminoglycoside and a penicillin or VAN in the treatment of infectious endocarditis (Baddour et al., 2015).

The data shown in Table 1 and Figure 2 also revealed that VAN/DSF displays synergistic killing of multiple MRSA strain types. Previously, we reported that DSF had a similar interaction with another antibiotic that disrupts staphylococcal cell wall synthesis (Frazier et al., 2019). Fosfomycin displayed synergy (ΣFIC 0.25) and enhanced bactericidal activity with DSF in fosfomycin-resistant JE2 (i.e., *fosB*^+^), which was attributed to drug-mediated reduction of FosB-dependent bacillithiol (BSH) (Lewis et al., 2022). This mechanism is not likely applicable to the VAN/DSF interaction, therefore other cell wall-related factors were considered for the increased killing action observed with the Mu50 strain.

Prior studies on Mu50 (Cui et al., 2000) and a VRSA isolate lacking either *vanA* or *vanB* genes to generate D-lactate terminating peptidoglycan precursors (Sieradzki and Tomasz, 1997) were shown to have increased VAN tolerance due to elevated levels of uncrosslinked muropeptides to entrap VAN near the cell wall surface. Our flow cytometry data supports the mechanism that VAN-treated VISA/VRSA have more muropeptide targets to raise the MIC of VAN. Figure 3 shows a higher level of VAN-BDP FL bound with VAN treatment at the 22 h time point compared to Mu50 cultures incubated with either vehicle, DSF, or VAN/DSF. The addition of DSF to VAN gave similar MFIs to untreated Mu50 suggesting that the combination did not induce muropeptide fortification to consume VAN. It is believed that the unaltered muropeptide composition with DSF co-treatment partly explains the increased killing effect of VAN within the first 24 h. By the 44 h time point, the MFI data suggests that the cellular influences of DSF subsided and VAN prompted the viable bacteria remaining to increase muropeptide levels for VAN-BDP FL to bind, increasing the fluorescence.

A final aim of the study was to define the influence of DSF on the PAE of VAN. Shorter periods were observed for Mu50 with DSF addition indicating an antagonistic interaction with VAN. The reason for the PAE differences between VAN and VAN/DSF is not yet evident, but the suppressed ATP production suggests it may be associated with metabolism. The VAN/DSF was found to impair ATP synthesis (Figure 1), which could result in the construction of fewer muropeptides for VAN to remain bound to and inhibit growth after removal from the media. This finding may have a translational implication requiring a modified interval dosing schedule of VAN due to a shorten PAE with DSF combined.

Additionally, clinical studies would need to evaluate potential side effects of the combination. Despite use as a maintenance therapy and low incidence of adverse effects in the absence of alcohol (Kalra et al., 2014), the extensive metabolism of DSF (Johansson, 1992; Custodio et al., 2022) may give rise to metabolites that could amplify parenteral VAN-associated toxicities (e.g., ototoxicity) and/or alter excretion rate. Translational studies will therefore need to delineate the PK parameters of the combination. Moreover, as a medication that was approved in the 1951 by the FDA, the clinical PK of DSF is poorly defined by modern standards (Johansson, 1992) and additional studies will establish its optimal use as an adjuvant for anti-infective agents.

In conclusion, this research indicates that DSF may be useful as an antibiotic adjuvant in VAN therapy for MRSA infections. Flow cytometry enabled confirmation of the increased killing effects for the combination, while providing evidence that DSF may counteract the VISA fortification mechanism to impede the action of VAN as an inhibitor of cell wall synthesis. Future objectives of this research will be to define how DSF alters metabolism in *S. aureus* using transcriptome data and conduct PK/PD studies in a VISA infection model. The goals of the *in vivo* experiments will be to establish the PK parameters of VAN/DSF treatments and determine if oral DSF potentiates parenteral VAN clearance of a MRSA infection. Moreover, the use of RNA-Seq differential expression analysis will facilitate the identification of regulated metabolic pathways altered by DSF to elucidate the mechanism(s) by which MRSA susceptibly to VAN increases when combined.

## Data availability statement

The original contributions presented in this study are included in the article, further inquiries can be directed to the corresponding author.

## Author contributions

TL: conceptualization, methodology, writing original draft, project administration, and funding acquisition. HC, YM, and TL: data curation, review, and editing. All authors have read and agreed to the published version of the manuscript.

## Funding

This research was funded by the National Institute of Allergy and Infectious Diseases, National Institutes of Health AI151970.

## Conflict of interest

The authors declare that the research was conducted in the absence of any commercial or financial relationships that could be construed as a potential conflict of interest.

## Publisher’s note

All claims expressed in this article are solely those of the authors and do not necessarily represent those of their affiliated organizations, or those of the publisher, the editors and the reviewers. Any product that may be evaluated in this article, or claim that may be made by its manufacturer, is not guaranteed or endorsed by the publisher.
