# Supplementary material for: Antimicrobial pharmacodynamics of vancomycin and disulfiram (Antabuse®) in Staphylococcus aureus
Source: Front Microbiol. 2023 Jan 6;13:1092257. doi: 10.3389/fmicb.2022.1092257 (PMC9854118; doi:10.3389/fmicb.2022.1092257)
Supplement: Supplementary file 1 [file Table_1.docx]

**SUPPLEMENTAL MATERIALS**

Antimicrobial Pharmacodynamics of Vancomycin and Disulfiram (Antabuse^®^) in *Staphylococcus aureus*

**Hasitha Chavva^a^, Yogesh Meka^a^, Timothy E. Long^a,b^[[1]](#footnote-1)^*^**

^a^ Department of Pharmaceutical Science and Research, School of Pharmacy, Marshall University, Huntington, WV, USA

^b^ Department of Biomedical Sciences, Joan C. Edwards School of Medicine, Marshall University, Huntington, WV, USA

| **Table 1S:** Strain and source information for bacteria used in the study. | | |  |  |
| --- | --- | --- | --- | --- |
| **Stain Designations** | **Characteristics** | **Source/Acknowledgement** | | |
| JE2, USA300 LAC, NR-46543 | Plasmid-cured derivative of USA300 MRSA strain LAC isolated from a skin and soft tissue infection of an inmate in the Los Angeles County Jail in California, USA in 2002. SCC*mec*: Type IV | Provided by the Network on Antimicrobial Resistance in Staphylococcus aureus (NARSA) for distribution through BEI Resources, NIAID, NIH: Staphylococcus aureus subsp. aureus, Strain JE2, NR-46543. | | |
| COL, NR-45906 | Hospital-acquired MRSA strain from the United Kingdom, SCC*mec*: Type II | Provided by the Network on Antimicrobial Resistance in *Staphylococcus aureus* (NARSA) for distribution by BEI Resources, NIAID, NIH: *Staphylococcus aureus*, Strain COL, NR-45906. | | |
| Mu3, NRS2, ATCC 700698 | hVISA strain with heterogeneous susceptibility to vancomycin obtained in sputum from lung cancer patient with MRSA pneumonia. SCC*mec*: Type II | Acquired from the American Type Culture Collection (Manassas, VA) | | |
| Mu50, NRS1, ATCC 700699 | VISA stain isolated from debridement tissue at surgical incision in sternum of an infant, SCC*mec*: Type II | Acquired from the American Type Culture Collection (Manassas, VA) | | |
| VISA AR-216 | Clinical isolate positive for aph(3')-III, aph-STPH, fosB, mecA, mph(C), sat-4A | Provided by Antimicrobial Resistance Isolate Bank for distribution by the CDC and FDA (Manassas, VA) | | |
| VISA AR-217 | Clinical isolate positive for aph-STPH, blaI, dfrG, fosB, mecA, Z | Provided by Antimicrobial Resistance Isolate Bank for distribution by the CDC and FDA (Manassas, VA) | | |
| VISA AR-218 | Clinical isolate positive for aph(3')-III, aph-STPH, DHA1, erm(A), mecA, norA, spc, tet(38), tet(K) | Provided by Antimicrobial Resistance Isolate Bank for distribution by the CDC and FDA (Manassas, VA) | | |
| VISA AR-219 | Clinical isolate positive for aac(6')-aph(2''), aadD, aph-STPH, DHA1, erm(A), mecA, norA, spc, tet(38), tet(K) | Provided by Antimicrobial Resistance Isolate Bank for distribution by the CDC and FDA (Manassas, VA) | | |
| VISA AR-220 | Clinical isolate positive for aph-STPH, DHA1, erm(A), mecA, norA, spc, tet(38) | Provided by Antimicrobial Resistance Isolate Bank for distribution by the CDC and FDA (Manassas, VA) | | |
| VRSA-MI, HIP11714, NR-46410 | USA100 MRSA strain isolated in 2002 in Michigan, USA from an infected catheter exit site of a adult who had a VRE co-infection and received a 6-week course of vancomycin. *SCCmec:* Type II and *vanA* positive. | Provided by the Network on Antimicrobial Resistance in *Staphylococcus aureus* (NARSA) for distribution by BEI Resources, NIAID, NIH: *Staphylococcus aureus*, Strain HIP11714, NR-46410. | | |

1. * Corresponding author: 1 John Marshall Drive, Huntington, WV, 25755; Tel.: +1-304-696-7393; fax: +1-304-696-7309; e-mail: [longt@marshall.edu](mailto:longt@marshall.edu) [↑](#footnote-ref-1)
